# Electronic voting to encourage interactive lectures: a randomised trial

**DOI:** 10.1186/1472-6920-7-25

**Published:** 2007-07-27

**Authors:** Paul M Duggan, Edward Palmer, Peter Devitt

**Affiliations:** 1 Discipline of Obstetrics and Gynaecology, Faculty of Health Sciences, The University of Adelaide, Adelaide, Australia; 2 Centre for Learning and Professional Development, The University of Adelaide, Adelaide, Australia; 3 Discipline of Surgery, Faculty of Health Sciences, The University of Adelaide, Adelaide, Australia

## Abstract

**Background:**

Electronic Voting Systems have been used for education in a variety of disciplines. Outcomes from these studies have been mixed. Because results from these studies have been mixed, we examined whether an EVS system could enhance a lecture's effect on educational outcomes.

**Methods:**

A cohort of 127 Year 5 medical students at the University of Adelaide was stratified by gender, residency status and academic record then randomised into 2 groups of 64 and 63 students. Each group received consecutive 40-minute lectures on two clinical topics. One group received the EVS for both topics. The other group received traditional teaching only. Evaluation was undertaken with two, 15-question multiple-choice questionnaires (MCQ) assessing knowledge and problem solving and undertaken as a written paper immediately before and after the lectures and repeated online 8–12 weeks later. Standardised institutional student questionnaires were completed for each lecture and independent observers assessed student behaviour during the lectures. Lecturer's opinions were assessed by a questionnaire developed for this study.

**Results:**

Two-thirds of students randomised to EVS and 59% of students randomised to traditional lectures attended. One-half of the students in the EVS group and 41% in the traditional group completed all questionnaires. There was no difference in MCQ scores between EVS and traditional lectures (p = 0.785). The cervical cancer lectures showed higher student ranking in favour of EVS in all parameters. The breast cancer lectures showed higher ranking in favour of traditional lectures in 5 of 7 parameters (p < 0.001). The observed higher-order lecturer-students interactions were increased in the EVS lecture for one lecturer and reduced for the other. Both lecturers felt that the EVS lectures were difficult to prepare, that they were able to keep to time in the traditional lectures, that the educational value of both lecture styles was similar, and that they were neutral-to-slightly favourably disposed to continue with the EVS technology. The 2 lecturers disagreed regarding the ease of preparation of the traditional lecture, their ability to keep to time in the EVS lecture, and personal satisfaction with the EVS lecture. The lecturers felt that EVS encouraged student participation and helped identify where students were having difficulty.

**Conclusion:**

In this setting, EVS technology used in large group lectures did not offer significant advantages over the more traditional lecture format.

## Background

The traditional, didactic lecture has been an integral part of many medical curricula. Traditional lectures have been used to transmit as economically as possible information and ideas, with the expectation that the students will somehow retain and use the material provided. Deficiencies in the outcome of this approach include a failure of students to demonstrate understanding of subject matter, as opposed to regurgitation of "facts", coupled with inadequate problem-solving skills and, perhaps, limited enthusiasm for the subject [1,2].

According to McCarthy and Anderson, "the traditional format [lecture] encourages students to concentrate on superficial indicators rather than on fundamental underlying principles.... Active learning refers to "experiences in which students are thinking about the subject matter" [3]. One way to encourage active learning in lectures is to employ an electronic voting system (EVS) (or audience response system) [4-9]. The EVS utilises wireless technology to permit students to respond anonymously to questions posed by the lecturer, thereby creating an interactive "low-risk" environment. Class responses can be graphically displayed during the lecture, which permits students to gauge where they are in relation to their peers. The time required to pose questions and obtain, display and discuss responses may require the lecturer to cover less material during the lecture but this may be a preferable method if one of the outcomes is deeper learning of the subject material.

The aim of this study was to examine the effect an electronic voting system (EVS) when used as an integral part of a lecture, in terms of cognitive outcomes, interaction and lecturer and student satisfaction.

## Methods

### Setting

This study was undertaken in the 2006 Common Program lecture series of the Year 5 MBBS program at the University of Adelaide, Australia.

Until 2004, most teaching in Year 5 of the 6-year undergraduate medical degree involved small groups or one-on-one clinical sessions. As a result of major curriculum changes teaching time for some disciplines was substantially reduced and, to partly compensate, the Common Program lecture series was introduced for all students. The lectures are scheduled in 3 hour blocks once weekly through the academic year. Lecturers raised concerns about poor student attendance and behaviour (e.g. inattention, use of mobile phones). The same students and teachers also participated in well-received small group teaching, suggesting that the problem with the lectures was either the content or the format. It was possible that some lecturers were not engaging the students because of a perception that as much material had to be imparted in the allotted time as possible, not allowing time for any interaction. In different settings Electronic Voting Systems (EVSs) have been used regularly by one or two individuals in the Medical School, but overall, there is a lack of familiarity and availability of the devices within the Faculty. This study reports the first use of an electronic Voting System (EVS) in the Common Program.

A cohort of 127 Year 5 MBBS students was stratified by gender and residency status (Australian or International) then randomised by academic ability (determined from the MCQ results of their Year 4 final examination undertaken in 2005) into 2 groups of 64 and 63 students. Each group received two 40-minute lectures on the topics of "Screening for Breast Cancer" and "Screening for Cervical Cancer". One group received two lectures delivered in the lecturer's usual format (called "Traditional") and the other group received the same material but in this case it had been structured to be interactive using an EVS. The lecturers were both experienced lecturers and specialists in their topic, had not used EVS before, and had not been instructed in detail as to how to present either the traditional lecture or the EVS lecture. The lecturers were required to present their lecture twice the same afternoon – once in EVS and once in Traditional format. The lecturers, who had lectured on these topics since the inception of the Common Program, were introduced to the EVS technology, offered a video example of two other lecturer's styles of traditional and EVS lecture, and asked to come up with their own material. The lecturers used 5 or 6 multiple-choice questions with 5 possible answers per question for discussion during each EVS session.

The lecturers who participated in this trial did not undergo any formal preselection and had not received teaching awards or any previous adverse evaluations.

Approval for the study was obtained from the University of Adelaide Ethics Committee.

### Outcomes

#### Cognitive skill

Cognitive skill was measured with two 15-question multiple-choice questionnaires (MCQ) covering the two lecture topics with one mark awarded for each correct answer and zero awarded for incorrect or missing responses. These marks were converted to a percentage. The questions tested knowledge and problem-solving ability in relation to clinical scenarios. These tests were administered as paper tests immediately before the lectures, again immediately after both lectures had finished, and 8–12 weeks later using an on-line examination system. Exactly the same test was administered on each occasion. Students who had not completed the questionnaire on-line by 8 weeks were sent electronic reminders weekly until 12 weeks.

#### Student opinion

Student opinion on the lectures was measured using a standardised University of Adelaide Student Experience of Learning and Teaching (SELT) questionnaire for each lecture. The survey consisted of 7 standard questions with Likert responses on a 7-point scale (range strongly disagree agree – strongly agree) and 2 open-ended questions for student comments [10].

#### Lecturer opinion

Lecturer opinion was canvassed using a questionnaire designed for this study. It consisted of 9 Likert response questions with a 7-point scale (range strongly disagree – strongly agree, questions 1–9 below) and 3 statements, numbered 10–12 below, seeking open-ended responses, as follows:

1. I found it easy to prepare the EVS lecture.

2. I found it easy to prepare the non-EVS lecture.

3. I was able to keep to time in the EVS lecture

4. I was able to keep to time in the non-EVS lecture

5. I was personally satisfied with the EVS lecture

6. I was personally satisfied with the non-EVS lecture

7. I felt the EVS lecture had high educational value

8. I felt the non-EVS lecture had high educational value

9. I will continue to use EVS in my lectures

10. What I liked about the EVS lecture compared with the non-EVS lecture:

11. What I disliked about the EVS lecture compared with the non-EVS lecture:

12. Other comments:

### Observation protocol

The behaviour and interactions between students was monitored in each of the teaching sessions by at least two independent observers. The observers had studied the protocol described elsewhere [9]. They had then practised using the methodology in other classes in order to develop their skills. At the end of each session, the observers compared notes to ensure consistency in observations. The results of the observer closest to the interaction were used where discrepancies arose. Table 1 shows the schema used to classify the interactions between students and lecturer. We considered a Level 2 or Level 3 interaction to be a meaningful interaction. This methodology has been used successfully in the past to measure student interactions [9].

**Table 1:** Schema for assessing levels of interaction

Level 1	Simple Interaction e.g. a yes/no answer to a question from a student or tutor
Level 2	A short answer (<1 min) to a question from either student or tutor
Level 3	An extended interaction of at least 1 minute creating further discussion

### Statistical analysis

**Figure 1:**
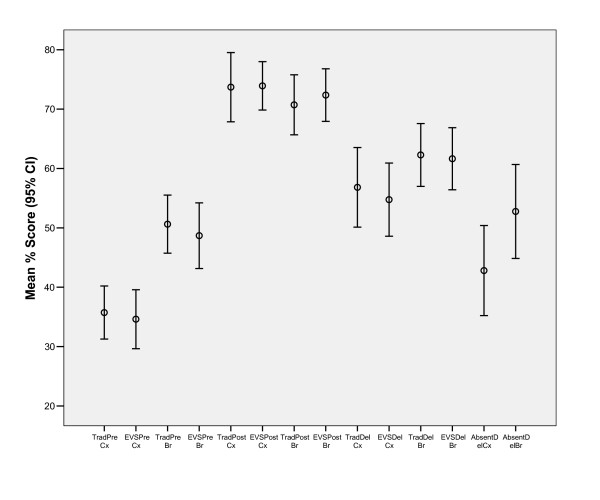
Mean percentage (95% confidence interval) values for the repeated multi-choice tests categorised by lecture type and topic. The differences between baseline, post- lecture and delayed testing are statistically significant (p = 0.037). The difference in baseline for the two topics is statistically significant (p < 0.001). The difference in delayed testing between attendees and absentees is significant (p < 0.001). Trad = traditional lecture; EVS = EVS lecture; Cx = cervical cancer topic; Br = breast cancer topic; Pre = test sat immediately before lecture; post = test sat immediately on completion of lectures; Del = delayed test 8–12 weeks after lecture; Absent = students who did not attend lectures but sat the delayed test.

Statistical analysis was undertaken with SPSS 13 for Windows. The MCQ results were analysed using the repeated measures General Linear Model, two-tailed t-test, Pearson correlation test and descriptive statistics. In the General Linear Model the MCQ results were treated as within-subject variables, and lecture type, gender and residency status as between-subject variables, and academic record as a co-variable. Secondary analyses were run using the two-tailed t-test comparing the 2005 academic year results of participating and non-participating students, and the results of the on-line MCQ comparing the results of students who had attended the lectures against those who had not. A power calculation was run in the General Linear Model and a power of 0.8 at the 0.05 significance level was considered to be adequate. The Pearson correlation test was run to correlate 2005 academic results with the results of the MCQ tests for completers of the tests. Simple descriptive statistics were also calculated. Data comparing the frequency of attendance at lectures and completion of the evaluation for the variables lecture type, gender and national status were analysed using the Chi-square test. The Likert responses from the SELT questionnaires were analysed using the Kruskal-Wallis test of ranks. The Kruskal-Wallis statistic measures how much the group ranks differ from the average rank of all groups. The graph of the MCQ results (Figure 1) was produced using the error bar option to display the mean and 95% confidence intervals.

A p-value of ≤ 0.05 was required for a statistical significance.

The Cronbach's alpha reliability coefficient was calculated for the SELT questionnaires and for the delayed (8–12 week) MCQ scores using the Scale – Reliability analysis feature in SPSS. Cronbach's alpha ≥ 0.8 was considered to indicate high reliability of the assessment.

The results of the observational protocol and lecturers' responses were described but not analysed for statistical significance.

### Ethical approval

Approval for this project has been obtained from the University of Adelaide Ethics Committee.

### Duration of the study

It was initially planned to run this study in lectures in April (as reported here), May and September of 2006, to involve 6 different lecturers from a range of disciplines, and to reverse the student allocations between EVS and traditional lectures in April and May and re-randomise in September. Unfortunately, our May lecturer volunteers, who had been recruited 6 weeks in advance, withdrew at very short notice citing excessive work commitments and lack of time to prepare an EVS lecture. We cancelled our plans to run the study again in September as we felt that students would be unlikely to attempt the delayed on-line MCQ test in November as this would clash with preparation for their major barrier examination at the end of the academic year.

## Results

From a cohort of 127 students, 43 students attended the EVS lectures and 38 students attended the Traditional lectures (67% versus 60% of those randomised, difference not significant, p = 0.8). The remaining members of the cohort were absentees. This level of absenteeism is not unusual for the Common Program. Twenty-nine students who attended the EVS lectures and twenty-six students who attended the traditional lectures fully completed the MCQ's including the delayed, on-line MCQ (45% versus 41% of those randomised, difference not significant p = 0.69). There was no difference in 2005 academic results between completing EVS and Traditional students (Mean (standard deviation) scores 56 (7) % versus 57 (6) %, respectively, p = 0.59).

There was no difference in the change in MCQ scores between baseline and at subsequent testing for students who had attended EVS or traditional lectures for either breast cancer or cervical cancer topics (p = 0.785, Figure 1). However, there was a significant difference in baseline MCQ scores between cervical cancer and breast cancer topics (p = 0.037). There was no difference in performance based on gender (p = 0.395) or residency status (p = 0.814).

The MCQ data (Figure 1) showed a large increase from baseline for both the immediate post-lecture test and the delayed post-lecture test for both topics (Figure 1). However, there was a significant decrease between the immediate post-lecture and delayed post-lecture MCQ scores, indicating a decay in cognitive skill over the 8–12 weeks that followed the lectures. In both cervical cancer and breast cancer topics the delayed results were slightly lower for the EVS group than the Traditional group (56.8 (17) % vs 54.8 (16) % and 62.3 (13) % vs 61.6 (14) %, respectively, p > 0.7). The power of this study to detect a difference of that level was low, estimated by the General Linear Model to be 0.1 (desired 0.8).

The MCQ results were significantly correlated with the students' MCQ score in the previous year's final examinations (Pearson correlation coefficient 0.43 for the cervical cancer topic and 0.53 for the breast cancer topic, p = 0.001). Absentees from the lectures and non-completers of this study were statistically more likely to be male (p = 0.02) or have Australian residency (p = 0.005) than those who completed the study.

A total of eighteen absentees from the lectures undertook the delayed on-line MCQ (Figure 1). Their mean (standard deviation) percentage scores were significantly lower for both topics than those for students who had attended lectures (43 (15) % versus 56 (17) % for the cervical cancer topic, p < 0.001 and 54 (15) % versus 62 (13) % for the breast cancer topic, p < 0.001). Attendees had higher 2005 academic scores than absentees (mean 56 (6) % versus 51 (6) %, p < 0.001) and students who fully completed the evaluation had higher 2005 academic scores than students who did not (mean 57 (6) % versus 53 (7) %, p = 0.004).

The Cronbach's alpha reliability coefficients for the on-line MCQ tests taken 8–12 weeks after the lectures were 0.58 for the cervical cancer topic and 0.28 for the breast cancer topic.

### Results of the SELT questionnaires

All students who attended the lectures completed the SELT questionnaire with a small number of missing variables (Table 2). The cervical cancer lectures showed higher ranking in favour of EVS in all 7 SELT domains. In contrast, the breast cancer lectures showed higher ranking in favour of Traditional lectures in 5 of 7 domains (p < 0.001). Column 4 of Table 2 shows the score for each domain expressed as a percentage of the maximum possible score allowing for missing values. This column is provided for information and is not part of the Kruskal Wallis calculation.

**Table 2:** Kruskal-Wallis analysis on ranks for the 7-item SELT questionnaire (range: strongly disagree-strongly agree, points value of range: 1–7, maximum possible score per item = Nx7). All differences for the 7 domains significant for the grouping variable "Lecture" (p < 0.001).

Domain	Lecture	N	Rank Order	Score expressed as % of maximum possible score (not used for Kruskal Wallis statistic)	Mean Rank Score for Kruskal Wallis statistic
Effectiveness	Trad Br	38	3	78	67
	EVS Br	40	4	74	54
	Trad Cx	43	2	86	94
	EVS Cx	43	1	90	111
					
Organisation	Trad Br	38	3	86	82
	EVS Br	41	4	74	45
	Trad Cx	43	2	91	100
	EVS Cx	43	1	91	104
					
Concern	Trad Br	38	3	81	78
	EVS Br	41	4	77	62
	Trad Cx	43	2	85	87
	EVS Cx	43	1	89	104
					
Enthusiasm	Trad Br	37	4	75	62
	EVS Br	41	3	77	65
	Trad Cx	43	2	84	85
	EVS Cx	43	1	93	115
					
Participation	Trad Br	37	4	71	59
	EVS Br	41	3	75	63
	Trad Cx	43	2	85	90
	EVS Cx	43	1	92	114
					
Stimulation	Trad Br	38	3	71	62
	EVS Br	41	4	69	61
	Trad Cx	43	2	82	93
	EVS Cx	43	1	89	113
					
Clarity	Trad Br	38	3	80	75
	EVS Br	41	4	69	49
	Trad Cx	43	2	87	94
	EVS Cx	43	1	91	111

Trad Br = traditional breast cancer lecture, EVS Br = EVS breast cancer lecture, Trad Cx = traditional cervical cancer lecture, EVS Cx = EVS cervical cancer lecture.

The Cronbach's alpha reliability coefficient for the SELT questionnaires were 0.94 for the cervical cancer lectures and 0.92 for the breast cancer lectures.

### Results of lecturer evaluations

The lecturers' opinions tended to agree for 5 of the 9 Likert responses. Both felt that the EVS lectures were difficult to prepare, that they were able to keep to time in the traditional lectures, that the educational value of both lecture styles was similar and not especially high, and that they were neutral to slightly favourably disposed to continue with the EVS technology. There was divergence in opinion regarding the ease of preparation of the traditional lecture, ability to keep to time in the EVS lecture, and personal satisfaction with the EVS lecture. In addition, the lecturers commented positively towards the EVS stating that it encouraged student participation, indicated where students were having difficulty, and showed which MCQ distractors needed to be rewritten. Negative comments towards EVS from one lecturer related to lack of familiarity with the operation of the system.

### Results of observational protocol

Data from the observational protocol are presented in Table 3. The sessions in which the EVS was used showed little student participation except at times when the EVS was used. A question asked by the lecturer, which required use of the EVS produced discussion at a class level on every occasion. Lecturer 1 (breast cancer topic) had no higher level interactions with students in his Traditional lecture and 5 higher order interactions in his EVS lecture, whilst lecturer 2 (cervical cancer topic), who used discussion techniques in engaging his class in the Traditional group, had 7 high level interactions in his Traditional lecture and 5 higher level interactions in his EVS lecture.

**Table 3:** Summary of independent observations during lectures

	No EVS Breast cancer topic	EVS Breast cancer topic	No EVS Cervical cancer topic*	EVS Cervical cancer topic
Minutes spent per lecture	50	37	45	45
Order of lecture in afternoon	2	1	1	2
% Students participating	40%	100%**	96%	100%**
Mean (standard deviation) no. students writing/5 minute interval	15 (3)	14 (10)	13 (6)	11(8)
%. students writing during lecture	77%	82%	89%	77%
No. level 1 interactions	0	3	6	4
No. level 2 interactions	1	3	4	5
No. level 3 interactions	0	2	3	0
No. student-student interactions	11	12	21	11

*This lecturer instigated discussion questions in the classroom. 33% of the student-student interactions occurred at this point.

**All students participated during the usage of the EVS. Very little participation occurred out of those times.

The students in the EVS class had extended periods where they were not writing, coinciding with the periods when the EVS was used. This is reflected in table 3 where the average number of students writing in any given 5 minute period varies little from lecturer to lecturer or by use of EVS, but the standard deviation becomes much larger. Overall, the percentage of students who wrote at some stage during the lecture was high (77%–89%) for both EVS and non-EVS lectures.

The observers noted that lecturer 2 had the greater technical knowledge and displayed greater comfort using the EVS system.

## Discussion

This study measured the effect of an electronic voting system on cognitive outcomes and classroom dynamics in lectures on two women's health topics. Students were randomised to either EVS or Traditional lectures based on contextually relevant, proven cognitive ability determined by their previous year's final MCQ examination results, after stratification for the important confounders of gender and residency status. We believe this randomisation process sets this and our previous study of small groups [9] apart from all other published studies of EVS and provides good generalisability. We found no difference between EVS and Traditional lectures in post-lecture MCQ results either immediately or at delayed testing 8–12 weeks later. There was a decay over time of similar magnitude in the results between the EVS and Traditional groups. A similar decay over time has been reported elsewhere [11]. EVS students scored slightly worse, on average by one or two percentage points, in the delayed testing compared to Traditional students, however the study was underpowered and this small difference was not statistically significant. The observed decay in results and the non-significant poorer performance of EVS students at delayed testing does not support the hope that EVS could promote higher order learning. A similar failure of EVS to enhance cognitive outcomes compared with traditional lectures was reported in an undergraduate nursing program, although the authors reported improved student satisfaction with EVS [12].

Others have shown improved cognitive outcomes, measured with MCQ's or short answer questions, with EVS compared to traditional formats in undergraduate lectures or small group teaching [4,7,8]. The differences between these studies and our own could be explained by bias introduced in other studies resulting from failure of adequate or any randomisation of subjects and the use of lecturers who were EVS enthusiasts. The papers cited are not from medical faculties and differences in the students may also account for some of this difference.

A small study of 17 postgraduate residents in Obstetrics and Gynaecology who were randomised to either a single EVS or traditional lecture on contraceptive choices reported improved cognitive outcomes assessed by a 15-question test administered immediately before and 6 weeks after the lectures [13]. There was no mention in this paper regarding controlling for the residents' cognitive abilities. It is not clear why the cited study and our own previous study of small groups [9] shows a cognitive benefit of EVS whereas the current study of EVS in a large lecture setting does not. We speculate that there may be another interaction other than EVS being observed in these small group settings, and that is likely to be the lecturer.

We found a significant correlation between the MCQ results in this study and our students' 2005 final examination results. Thus, a major determinant of outcome could be students' innate ability, which is independent of the style of lecture provided. The lack of any difference in MCQ scores between EVS and Traditional groups could also be explained if the questions used were not adequate to measure an effect of the EVS. We acknowledge that it is difficult to write MCQ's that test deep learning and that this is a potential weakness of most studies that use MCQ's. However, the style of MCQ we used, which required factual recall and the ability to interpret data or clinical scenarios, was identical to that used in our students' final examinations, and is a valid tool of relevance to our students.

Our observational protocol has been previously described [9]. This protocol records student interaction using an arbitrary scale based on the degree of interaction observed by trained observers. The highest level of interaction exceeds one minute and creates further discussion. This is indirect evidence for deep learning. The number of level 3 interactions was very small and on this analysis there is no convincing evidence of deep learning in action in either type of lecture. It is not known whether this is a true reflection of the (low) educational value of the lectures or whether the protocol that we have developed is unable to measure deep learning satisfactorily.

Satisfaction is commonly measured by questionnaires with a Likert scale such as the standard University of Adelaide SELT questionnaire [10]. The SELT showed variation between the 2 lecturers, with one lecturer being more successful with EVS and the other more successful with the traditional lecture. The lecturer who was more successful with EVS used student-teacher interaction as part of his usual "Traditional" lecture and despite this scored better on the SELTs for his EVS lecture. We speculate that the value of EVS may be primarily determined by the personal attributes of the lecturer.

Less than half of our eligible student body completed this study. Reanalysis of academic records for completers showed no significant differences between Traditional and EVS groups. We conclude that the randomisation was effective in maintaining an equivalent level of student ability between the two types of lecture despite the high rate of absenteeism and non-completion.

Absenteeism is not unusual in our Common Program and appears to be an international problem where there is evidence that absentees underachieve in final examinations [14,15]. The difference in 2005 academic record between our attendees and absentees and the poorer performance by absentees in the delayed MCQ supports this. We believe most absentees chose not to attend due to lack of interest in the topics, or a strategic choice to study the topics in some other way (e.g. expecting on-line lecture notes to become available or learning from textbooks or colleagues). Our absentees were statistically more likely to be male and hold Australian residency. The topics under evaluation were related to women's health and this may in part explain this gender bias. It is also possible that some students would have been put off by the research nature of the exercise.

Our students had better baseline knowledge of breast cancer screening because they had greater prior exposure to the breast cancer topic during their 4^th ^year clinical attachments, whereas few had completed their 5^th ^year gynaecology attachment at the time of this study. The gynaecology lectures introduced completely new material resulting from a nation-wide change in screening protocols for cervical cancer in 2006, so some of our students' previous knowledge was out of date, thus contributing to a poorer baseline MCQ performance.

Important limitations of this study include the use of only 2 lecturers for two topics and the relatively poor student completion rate. Two of our lecturers who had originally agreed to participate withdrew at short notice when they realised that preparing an EVS lecture would impose a substantially greater burden than they had first envisaged. Whilst anecdotal, this attitude amongst Faculty staff is likely to contribute to the poor utilisation of this and similar teaching aids.

The reliability of our delayed MCQ tests was very low for the breast cancer topic and low-moderate for the cervical cancer topic. Reliability would be improved with a larger number of questions and larger number of participating students. We felt that 15 questions was the maximal number that could be written for a single lecture topic and answered by the students within the available time. Reliability statistics have not been published in the other papers cited to enable a comparison.

To get the most from an EVS, users should be able to exploit its data collection and analysis capabilities, but the most important facet of any EVS system is likely to be the ability to provide immediate feedback to both teacher and student. Lecturers should be able to use this to gauge students' understanding and to adjust the lecture accordingly. This advantage was noted by one of our lecturers in his report (data not shown). It does, however, require additional flexibility from the lecturer and the confidence to depart from a prepared lecture, which in part demands a wide knowledge of the subject matter. EVS allows students to test their knowledge and skills in an anonymous, risk-free environment and be aware of where they stand in relation to their peers. Thus, the EVS is a type of formative assessment. Two of our students commented on the value of formative assessment in the EVS lectures in free comments on their SELT forms (data not shown).

Since this trial EVS has been adopted as the preferred method by one of the 2 participating lecturers with good results as measured by the SELT questionnaire. However, the EVS remains little-used throughout our institution, despite this study and others carried out previously.

Would the authors recommend an EVS system to the uninitiated? The answer is a qualified "yes". Lecturing with EVS is best suited to those who are confident with computer-based technology, willing to deviate from the prepared path as determined by student EVS responses, and able to put a lot of time in to developing interactive lectures. It may be that for these reasons, areas will dabble with the technology and then abandon it.

We undertook this study partly to establish if there was justification for adopting EVS delivery in our Common Program lectures, which would require a significant investment of funds and lecturers' time. We, in common with other medical faculties, are currently engaged in a debate over style versus content in delivery of the curriculum. Our data would not persuade colleagues who feel content is being underemphasised to adopt EVS delivery of lectures.

## Conclusion

EVS encouraged active learning as assessed by student-lecturer and student-student interactions but this was only apparent when questions were being posed during the EVS lecture and did not result in spontaneous interactions. An EVS lecture requires a substantial investment of lecturer's time and the effectiveness of the EVS as measured by student and lecturer opinion depends on the lecturer's ability to exploit the EVS technology. The strongest predictor of student performance in our MCQ's was student performance in the previous year's final MCQ examination. There was no difference in topic-related MCQ scores for students attending EVS or Traditional lectures and by this measure there is no learning advantage of the EVS over traditional lectures.

## Competing interests

The author(s) declare that they have no competing interests.

## Authors' contributions

EP participated in the design of the study, developed the observational protocol and helped to draft the manuscript. PD participated in the design of the study. PMD conceived of the study, and participated in its design and coordination, performed the statistical analysis and drafted the manuscript. All authors read and approved the final manuscript.

## Pre-publication history

The pre-publication history for this paper can be accessed here:

http://www.biomedcentral.com/1472-6920/7/25/prepub
